# Supplementary figures and images for: Swapping Metagenomics Preprocessing Pipeline Components Offers Speed and Sensitivity Increases
Source: mSystems. 2022 Mar 16;7(2):e01378-21. doi: 10.1128/msystems.01378-21 (PMC9040843; doi:10.1128/msystems.01378-21)

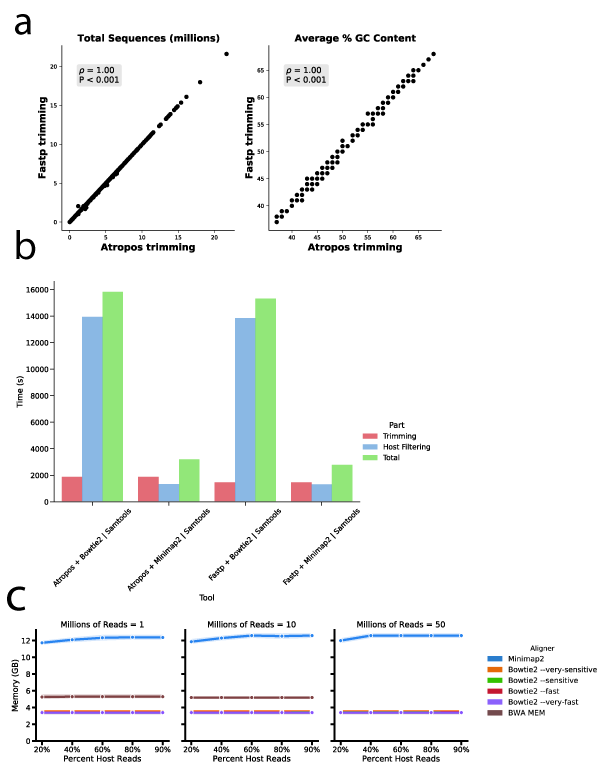

Supplement: FIG S1 [file msystems.01378-21-sf001.tif]

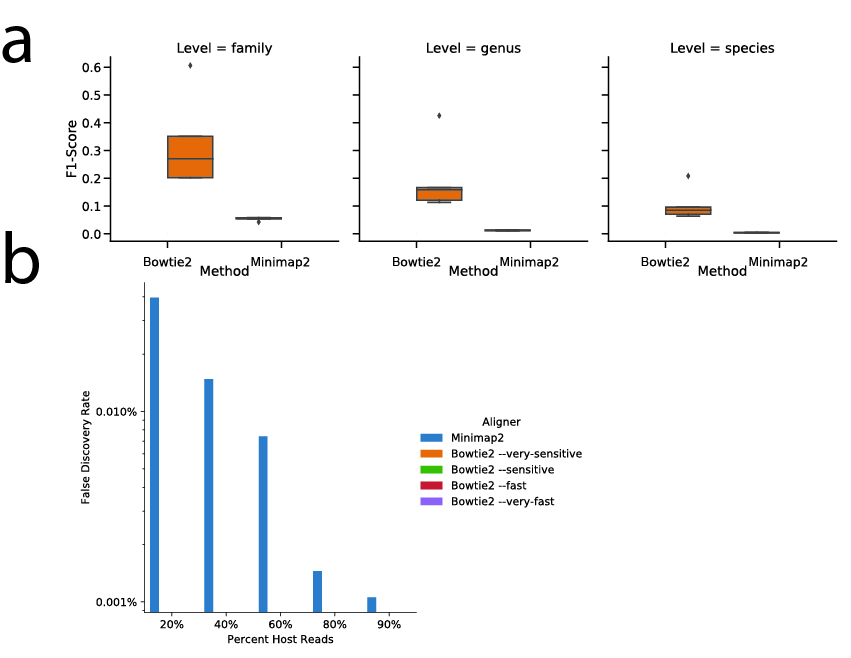

Supplement: FIG S2 [file msystems.01378-21-sf002.tif]
