# Supplementary material for: Swapping Metagenomics Preprocessing Pipeline Components Offers Speed and Sensitivity Increases
Source: mSystems. 2022 Mar 16;7(2):e01378-21. doi: 10.1128/msystems.01378-21 (PMC9040843; doi:10.1128/msystems.01378-21)
Supplement: TEXT S1 [file msystems.01378-21-s0001.docx]

1. Supplemental methods
   1. tool version numbers
      1. Atropos (v. 1.1.24)
      2. Bowtie2 (v. 2.3.2)
      3. Minimap2 (v. 2.17)
      4. Fastp (v. 0.20.1)
      5. bwa (v0.7.17)
   2. commands for alignment, simulation, evaluation in fig. 1 and woltka
      1. Simulation data:
         1. Simulated read genome accessions:
            1. GCF_000001405.39
            2. GCF_000009045.1
            3. GCF_000196035.1
            4. GCF_000013425.1
            5. GCF_000415185.1
            6. GCF_000010145.1
            7. GCF_000195995.1
            8. GCF_000008865.2
            9. GCF_000006765.1
         2. Alignment reference:
            1. GCA_009914755.3
         3. Command for read error simulation:
            1. art_illumina --len 150 --seqSys HS25 --noALN -i {filename} -o {output_filename} –fcov 3
         4. indexing
            1. time minimap2 -ax sr -d ref.mmi ref.fa
            2. time bowtie2-build ref.fa ref
            3. time bwa index ref.fa
         5. alignment:
            1. time minimap2 -t 12 -ax sr ref.mmi reads.fq > out.sam
            2. time bowtie2 --threads 12 --very-fast -x ref -U reads.fq -S out.sam
            3. time bwa mem ref reads.fq > out.sam
         6. memory:
            1. pmap -x $(pidof minimap2)
            2. pmap -x $(pidof bowtie2-align-s)
            3. pmap -x $(pidof bowtie2-build-s)
            4. pmap -x $(pidof bwa)
      2. Exome simulation data:
         1. Command for atropos and bowtie2
            1. alignment reference: GCF_000001405.39 with phiX174
            2. atropos trim -a GATCGGAAGAGCACACGTCTGAACTCCAGTCAC -A GATCGGAAGAGCGTCGTGTAGGGAAAGGAGTGT -q 15 --minimum-length 100 --pair-filter any --nextseq-trim 30 --threads $NPROCS --report-file ${filename1_short}.log --report-formats txt -o ${filename1_short}.trimmed.fastq.gz -p ${filename2_short}.trimmed.fastq.gz -pe1 $filename1 -pe2 $filename2
            3. bowtie2 run on --very-sensitive by default but also compared by --very-fast, --fast, and --sensitive

bowtie2 -p $NPROCS -x $dbbt2 --very-sensitive -1 ${filename1_short}.trimmed.fastq.gz -2 ${filename2_short}.trimmed.fastq.gz | samtools fastq -@ $NPROCS -f 12 -F 256 -1 $f1.bowtie2.filt.fastq -2 $f2.bowtie2.filt.fastq

- - - 1. Commands for fastp and minimap2
         1. fastp --adapter_sequence GATCGGAAGAGCACACGTCTGAACTCCAGTCAC --adapter_sequence_r2 GATCGGAAGAGCGTCGTGTAGGGAAAGGAGTGT -l 100 -i $filename1 -I $filename2 --stdout -w $NPROCS -h qc/${filename1_short}.html | $minimap2 -ax sr -t $NPROCS $db - -a | $samtools fastq -@ $NPROCS -f 12 -F 256 -1 $final_output/${filename1_short}.trimmed.fastq.gz -2 $final_output/${filename2_short}.trimmed.fastq.gz
    1. Woltka (v. 0.1.3) commands are default please see <https://github.com/qiyunzhu/woltka>
       1. Database: WoL
       2. Dataset: Five synthetic datasets generated using CAMISIM v1.1.0, each simulated from 10 genomes randomly chosen from WoL, with a lognormal distribution (mu = 1, sigma = 2) of abundances, followed by generation of 0.667 million pairs of 150 bp synthetic Illumina sequences.
       3. Bowtie2: default parameters: `bowtie2 -p 32 -x $db --interleaved input.fq.gz -S output.sam --seed 42`
  1. CPU details:
     1. Data access
        1. Architecture: x86_64
        2. CPU(s): 12
        3. Thread(s) per core: 2
        4. Core(s) per socket: 6
        5. Model name: Intel(R) Core(TM) i7-8700 CPU @ 3.20 GHz
        6. CPU max: 4.60 GHz
        7. L1d cache: 32K
        8. L1i cache: 32K
        9. L2 cache: 256K
        10. L3 cache: 12288K
        11. RAM: 32GB
        12. Disk: 2 TB SSD
        13. Ubuntu 18.04.5 LTS

1. Data Access
   1. Kit comparison data
      1. European Nucleotide Archive accession number ERP124610
      2. Qiita study identifier 12201
   2. Human exome data
      1. ENA project numbers PRJEB31736 and PRJEB36890
   3. Mouse microbiome metagenomics
      1. ENA under accession ERP116718
      2. Qiita study identifier 10537
